# Self-Emulsifying Phospholipid Preconcentrates for the Enhanced Photoprotection of Luteolin

**DOI:** 10.3390/pharmaceutics14091896

**Published:** 2022-09-07

**Authors:** Yun-Shan Hsieh, Yih-Fung Chen, Yung-Yi Cheng, Wan-Yi Liu, Yu-Tse Wu

**Affiliations:** 1 School of Pharmacy, Kaohsiung Medical University, Kaohsiung 80708, Taiwan; 2 Graduate Institute of Natural Products, College of Pharmacy, Kaohsiung Medical University, Kaohsiung 80708, Taiwan; 3 Division of Pharmacoengineering and Molecular Pharmaceutics, Eshelman School of Pharmacy, University of North Carolina, Chapel Hill, NC 27599, USA

**Keywords:** luteolin, self-emulsifying phospholipid preconcentrates, photoprotection

## Abstract

Exposure to ultraviolet B (UVB) leads to the overproduction of reactive oxygen species (ROS), causing higher risks of skin disorders. Luteolin (Lut) is a naturally occurring antioxidant that can absorb a broad range of ultraviolet light, but its water solubility and skin permeability are limited and insufficient. The aim of the current study was to develop a Lut-loaded self-emulsifying phospholipid preconcentrate (LSEPP) for enhancing the solubility, permeability, and photoprotective activity of Lut. The designed formulations were firstly examined for their droplet size, zeta potential, dispersity, and in vitro corneum permeability after dispensing the preconcentrate to form an emulsion; the optimized formulation was further characterized for its emulsified morphology, compatibility with excipients, stability in the preconcentrate form, and photoprotective activity by the HaCaT cell model under the emulsified status. The optimized LSEPP formulation attained a smaller droplet size (140.6 ± 24.2 nm) with the addition of 1,8-cineole and increased the permeability of Lut by 7-fold. As evidenced in the cell model studies, the optimized LSEPP formulation can efficiently deliver Lut into HaCaT cells after emulsification and result in a 115% better cell viability as well as a 203% stronger ROS scavenging capability, compared with those of unformulated Lut after UVB irradiation. To sum up, we have successfully developed an LSEPP formulation, which is a safe and promising topical delivery system for enhancing the photoprotective effects of Lut.

## 1. Introduction

Sunlight includes ultraviolet (UV) light, visible light, and infrared light. UV accounts for half of the sunlight and causes the most direct and severe damage to human skin [1]. A recent epidemiological study [2] conducted in the US has indicated that the prevalence of skin cancers in states with a high UV (11.9%) index is significantly higher (*p* < 0.0001) than those in states with medium (9.0%) and low (7.8%) UV indexes. Specifically, UVA (320–400 nm) and UVB (290–320 nm) are the major types of UV that cause severe skin damage [3]. Though the ratio of UVB is relatively low (10%) compared to that of UVA (90%), UVB has a much higher energy than UVA because of the short wavelength, and the probability of UVB-induced erythema is 1000-fold higher than UVA-induced erythema. UVB penetrates the stratum corneum to reach the epidermis, causing direct DNA damage, inflammation response, and ROS overproduction. These free radicals severely impair cell lipids, proteins, and genes to cause high risks of skin cancers [4]. Recently, researchers have found many natural compounds with photoprotective potential, such as polyphenols, flavonoids, and carotenoids. Among these compounds, flavonoids attract the most attention due to their broad and strong UV absorption and antioxidation and the enhancing capability of the Nrf2 pathway to accelerate the translation of antioxidative enzymes [5,6].

**Figure 1:**
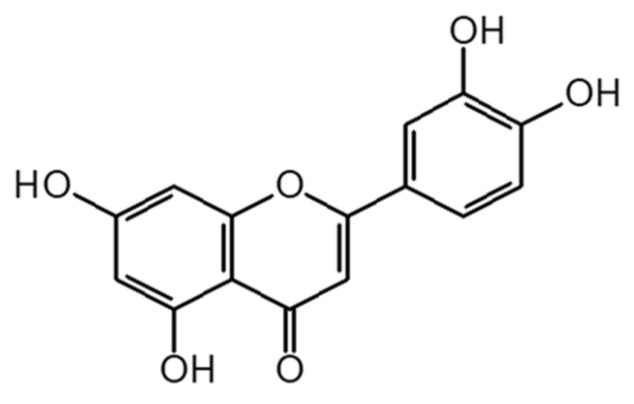
The structure of Luteolin.

Luteolin (Lut), 3′,4′,5,7-tetrahydroxyflavone, is a flavonoid, and its structure is illustrated in Figure 1. Lut possesses a broad UV absorption spectrum in 270–390 nm, which means it could attenuate the radiation from UV, and Lut also has notable antioxidative ability. According to the structure–antioxidant–activity relationship, the catechol group in the B ring could donate the hydrogen to reactive radicals as the terminator of the free radical chain, and the 1,4-pyrone moiety in the C ring allows unpaired electrons across the whole molecule to delocalize. When the newly formed flavonoid radicals are delocalized, they are less aggressive than free radicals [7,8]. Moreover, Lut can selectively protect normal human keratinocytes from UVB damage, and this activity is not observed in the malignant skin cells, which is an essential trait as a photoprotective agent [9]. Though Lut exhibits promising pharmacological bioactivities, the poor aqueous solubility of Lut in water (0.0055 mg/mL at 20 °C) limits the diffusion efficacy into skin [10]. Even though Lut has passes the stratum corneum, it may be kept out of cells owing to the high affinity to the cell membrane [11]. To improve permeability and cellular uptake, formulation design is necessary.

Only a few Lut-loaded formulations for skin delivery have been proposed to achieve desired permeability and better pharmacological effects. Shin et al. [12] developed a nanoemulsion for carrying Lut into hair follicles, which is the only Lut formulation that had been used in topical delivery until now. In addition, a phospholipid complex [13] and non-ionic surfactant-based vesicles [14] have been proposed for transdermal delivery of Lut to alleviate inflammatory diseases. However, Lut-loaded topical formulations for photoprotection are still absent nowadays. Therefore, self-emulsifying phospholipid preconcentrates (SEPPs) for topical delivery of Lut are proposed in this study.

SEPPs can be considered a subclass of a self-emulsifying drug delivery system, which is a micro/nanoemulsion preconcentrate consisting of an oil phase, surfactants, and cosurfactants [15,16]. SEPPs were designed for oral use initially but have been developed for topical use in recent years [17]. As SEPPs are preconcentrates, the drug loading could be much higher and have a greater stability than other formulations containing water. Users should add water to emulsify SEPPs before use, and they could adjust the amounts of water for the emulsification to apply on either small or huge areas with a comfortable sensory experience. In this study, a phospholipid was utilized as the oil phase. When emulsified, the phospholipid could self-assemble into the lipid bilayer to form droplets because of its shape and amphiphilic characteristics [18]. This delivery system also provides better skin occlusion and a higher degree of hydration for enhanced permeation [19]. Terpenes are well-known permeation enhancers and could be classified into different classes based on their structures. A monoterpene (1,8-cineole) and a sesquiterpene (nerolidol) were incorporated into luteolin-loaded SEPPs (LSEPPs), and their abilities in permeability enhancement were evaluated. The present study designed and evaluated the LSEPPs formulation, aiming to increase the drug loading to enhance permeation over the stratum corneum and to boost the photoprotective activity of Lut.

## 2. Materials and Methods

### 2.1. Materials

Luteolin [2-(3,4-dihydroxyphenyl)-5,7-dihydroxychromen-4-one, CAS#491-70-3)] with a purity >98%, Tween^®^ 80 (Polyoxyethylene 20 sorbitan monooleate, CAS#9005-65-6), and Span^®^ 80 [(Z)-Sorbitan mono-9-octadecenoate, CAS#1338-43-8] were purchased from Tokyo Chemical Industry (Tokyo, Japan). Castor oil, jojoba oil, and shea butter were obtained from Magie Fairy (Taichung city, Taiwan). PHOSAL^®^ 50 PG (Phosphatidylcholine in propylene glycol, content ≥ 50.0%) was obtained from LIPOID^®^ GmbH (Ludwigshafen, Germany). Kolliphor^®^ EL (Polyethoxylated castor oil, CAS#61791-12-6) was supplied by BASF (Ludwigshafen, Germany) and Tween^®^ 20 (Polyoxyethylene 20 sorbitan monolaurate, CAS#9005-64-5) was from Sigma-Aldrich Co. (St. Louis, MO, USA). 1,8-cineole (1,3,3-trimethyl-2-oxabicyclo [2.2.2]octane, CAS# 470-82-6) and nerolidol [(6E)-3,7,11-trimethyldodeca-1,6,10-trien-3-ol, CAS# 7212-44-4)] were purchased from Alfa Aesar (Haverhill, MA, USA). The MultiScreen^®^ Permeability Filter Plate, 0.4 µm, non-sterile (The skin PAMPA kit) was from Merck (Darmstadt, Germany). All solvents used in this study were of analytical grade.

### 2.2. Solubility Assessment of Lut

The solubility of Lut in oils (castor oil, jojoba oil, shea butter, and PHOSAL^®^ 50 PG) and emulsifiers (Span^®^ 80, Kolliphor^®^ EL, Tween^®^ 80, and Tween^®^ 20) was assessed. An excess amount of Lut was added to oils, then vortexed for 5 min and sonicated for 20 min. The mixtures were shaken in a thermostat water bath at 37 ± 0.5 °C and 50 rpm for 48 h to reach equilibrium. After that, these mixtures were centrifuged twice at 20,000× *g* for 15 min to remove undissolved Lut completely [20]. The 50 mg supernatants were extracted by 2 mL isopropanol and vortexed for 30 s. Next, 2 mL methanol was added and then vortexed for 30 s. The solution was filtered with 0.22 µm PTFE filters and subjected to HPLC-PDA analysis.

In the aspect of surfactants, an excess amount of Lut was added to surfactants, followed by the same step as in the previous paragraph. Mixtures were centrifuged twice at 13,600× *g* for 15 min and diluted to an appropriate concentration with 50% acetonitrile (ACN). Before HPLC-PDA analysis, the diluted solution was vortexed for 5 min and centrifuged for 15 min at 13,600× *g* prior to HPLC-PDA analysis.

The HPLC-PDA analysis system consists of an LC-10AT pump SHIMADZU, a SIL-10AF autosampler, and an SPD-M10A detector (Shimadzu Scientific Instruments, Kyoto, Japan). The column was a Kinetex^®^ C18 column (250 × 4.6 mm, 5 µm, Phenomenex, Inc., Torrance, CA, USA). The composition of the mobile phase was ACN: deionized water = 20:80 (*v*/*v*) and the flow rate was 1.0 mL/min. Samples of 20 μL would be injected and monitored at 343 nm. The formula of the calibration curve were y = 31959x − 2591.7 (R^2^ = 0.9999) and y = 41716x − 34150 (R^2^ = 0.9992), with a linear range from 0.25 to 5 µg/mL and from 5 to 50 μg/mL, respectively. The method accuracy and precision were within 10%, and the limit of quantification was 0.25 μg/mL.

### 2.3. Preparation of LSEPPs

The LSEPPs was composed of PHOSAL^®^ 50 PG, Kolliphor^®^ EL, and Tween^®^ 20 as the oil phase, emulsifier, and co-emulsifier, respectively. To optimize the ratio of the three components, various formulations were evaluated. The compositions of all formulations are listed in Table 1. The preparation procedures of LSEPPs have been modified appropriately from previous studies [15,21]. Briefly, excipients were blended into a 3 g mixture in total, and Lut (90 mg) was then incorporated into it. The resulting mixture was warmed and stirred at 50 °C for 1 h, which was then sonicated for 30 min to obtain yellow-colored LSEPPs with transparent appearance.

To further improve the permeation, two terpenoids (1,8-cineole or nerolidol) as enhancers were added to the optimized formulation. The design of F7 and F8 were based on the composition of F5. Therefore, F5 was blended with 1,8-cineole and nerolidol in a ratio of 95:5 (*w*/*w*) to give F7 and F8, respectively. The mixture was stirred for 15 min and sonicated for 15 min under ambient temperature to produce the final products.

### 2.4. Formulation Optimization

#### 2.4.1. Droplet Size, Polydispersity Index (PDI), and Zeta-Potential

The average droplet size, polydispersity index (PDI), and zeta-potential of the formulations were evaluated with dynamic laser scattering (ELSZ-2000, Otsuka Tech Electronics Co., Osaka, Japan). Each formulation was diluted 100 times with distilled water 10–15 min before the evaluation, and these characteristics were determined.

#### 2.4.2. Dispersity Test

Each LSEPPs was diluted 20-fold with a pH 6.4 phosphate buffer to simulate the skin environment, and the time taken for complete emulsification and the appearance were recorded. Based on the emulsification time and appearance, formulations were classified into five grades. Grade C exhibited a slow emulsification to form an opaque emulsion, and the properties implied the appropriate water resistance and better skin occlusion in topical delivery. As a result, Grade C is regarded as optimal for topical uses. The emulsification times for grade C and D were less and more than 2 min, respectively, and the appearance of grade C or D was milky white [17].

#### 2.4.3. In Vitro Permeation Study

A skin Parallel Artificial Membrane Permeability Assay (skin PAMPA) model was applied to evaluate the permeability in the stratum corneum, and the method was modified based on the manufacturer guide and a previous study [22]. Briefly, 200 µL 5% DMSO pH 7.4 PBS solution was transferred into each well of the receptor plate (the bottom layer). The donor plate was set up with the receptor plate. Thereafter, the donor plate (the top layer) was coated with 17 µL of the artificial liquid membrane composed of 65 wt% *n*-hexane and 35 wt% (isopropyl myristate/silicone oil, 3/7, *w*/*w*). Next, 300 µL of 1 mg/mL emulsified LSEPPs, which was dispensed with receptor solution 10–15 min before the experiment, was added to the donor plate. Sampling points were 1, 2, 4, and 6 h. UV/Vis spectroscopy was adopted as the analytical method. A SpectraMax iD3 (Molecular Devices, San Jose, CA, USA) was utilized; the detected mode was the endpoint, and the wavelength was 343 nm. The samples in plates would be shaken in an orbital mode for 10 s before analysis. The formula of the calibration curves were y = 0.0331x + 0.00003 (R^2^ = 0.9994) and y = 0.0293x + 0.0579 (R^2^ = 0.9992) with linear ranges of 0.25–10 μg/mL and 10–100 μg/mL, respectively, and the accuracy was within 100 ± 10%. The limit of quantification was located at 0.25 μg/mL.

### 2.5. Characterization

#### 2.5.1. Morphology

TEM was applied to observe the morphology of the optimized formulation. The formulation was dispensed 10–15 min before the experiment with deionized water and dropped on a 300-mesh copper grid coated with carbon. Consequently, it was stained with 2% (*w*/*v*) phosphotungstic acid solution (PTA), and the excess PTA was removed with deionized water. The stained grid was dried overnight and subjected to JEM-1400 Transmission Electron Microscope (JEOL, Ltd., Tokyo, Japan) at 120 kV [23].

#### 2.5.2. Compatibility between Lut and Excipients

To clarify the interaction between Lut and excipients, FTIR was applied to ensure the structure of Lut did not change, which may cause the loss of activity. The FTIR system was FT/IR-6800 (JASCO, Inc., Tokyo, Japan). Lut and KBr were dried overnight and mixed in a 1:100 (*w*/*w*) ratio, followed by grinding and compressing into a tablet. Excipients and F7 were analyzed by ATR, and air was used as the control group. The range of wavelength was evaluated at 600–4000 cm^−1^.

#### 2.5.3. Stability Evaluation

The optimized formulation was subject to heating–cooling and freeze–thaw cycles and a centrifugation test to observe if there were any signs of instability, including phase separation or drug precipitation. The heating–cooling cycles were conducted between 4 °C and 45 °C three times. Each temperature lasted no less than 24 h, and the freeze–thaw cycles were carried out between −20 °C and 25 °C with a storage period longer than 24 h at each temperature for three cycles [24]. In the centrifugation test, the formulation was centrifuged at 3000× *g* for 30 min [25]. The Lut content was determined after these cycles by UV/Vis spectroscopy after appropriate dilution.

### 2.6. Cell Viability

#### 2.6.1. Cell Culture

Human epidermal keratinocyte HaCaT cells were purchased from AddexBio (San Diego, CA, USA). The HaCaT cells were cultured in Dulbecco’s Modified Eagle Medium (DMEM) with 4.5 g/L glucose, 2 mM G-glutamine, 100 U/mL Penicillin, 100 µg/mL streptomycin, 0.25 µg/mL amphotericin B, and 10% fetal bovine serum (Sigma-Aldrich, St. Louis, MO, USA). Cells were maintained at 37 °C in a humidified incubator with 5% CO_2_. The culture medium was replaced every 2–3 days, when the cell reached 70–80% confluence.

#### 2.6.2. AlamarBlue^®^ Assays

The cell viability was determined by AlamarBlue^®^ assays. The cells were seeded into 96-well plates at a density of 1 × 10^4^ cells per well for 24 h when 70% confluence was achieved. The raw Lut was dispersed in DMEM, and LSEPPs and SEPPs were dispensed with DMEM 10–15 min before the experiment. All samples were added into 96-well plates; then, the cells were incubated for the indicated times. AlamarBlue^®^ reagent was added into the culture medium 2 h earlier than the indicated time and incubated in a cell incubator. The fluorescence with an excitation wavelength of 560 nm and an emission of 590 nm was read by using a SpectraMax iD3.

### 2.7. Cellular Uptake

To evaluate the cellular uptake of SEPPs, DiI-loaded SEPPs was prepared. The preparation method of DiI- loaded SEPPs was slightly modified from a previous study [26]. 1,1′-Dioctadecyl-3,3,3′,3′-tetramethylindocarbocyanine perchlorate (DiI C18(3), Invitrogen^TM^, Thermos Fisher Scientific, Waltham, MA, USA) was dissolved in ethanol to form 10 mg/mL stock solution. An 18 µL aliquot of DiI C18(3) was added into PHOSAL^®^ 50 PG to label phospholipid, and the preparative procedures were the same as described in Section 2.3. Preparation of LSEPPs, to prepare blank DiI-loaded SEPPs, followed by emulsification in DMEM. The HaCaT cells were cultured in a 96-well plate at a 1 × 10^4^ density overnight at 37 °C with 5% CO_2_; then, the emulsified DiI-loaded SEPPs were pretreated for 3, 6, and 24 h. At the predetermined time, cells were washed with PBS and fixed with 4% (*v*/*v*) formaldehyde for 15 min. After fixation, cells were washed with PBS again, and cell nuclei were stained utilizing Hoechst 33,258 solution (10 µg/mL) for 30 min. Finally, the cells were washed with PBS and kept at 4 °C. The results were imaged by ImageXpress^®^ Micro Widefield High Content Imaging System (Molecule Devices, San Jose, CA, USA).

### 2.8. Photoprotective Effects

#### 2.8.1. Cell Viability after UVB Irradiation

The procedures and conditions of cell culture, plating, and sample preparation are the same. The LSEPPs were pretreated for 6 h in a cell incubator. The cells were washed and incubated with PBS for UVB irradiation (CL-1000M UV crosslinker, UVP, Upland, CA, USA). The energy of UVB irradiation was 20 mJ/cm^2^ with a UV peak at 302 nm. After exposure, the cells were incubated in a fresh medium without samples in the incubator for 24 h, and their viability was analyzed as Section 2.6.2. AlamarBlue^®^ Assays described. Quercetin and all-*trans* retinoic acid were chosen as positive control owing to their excellent antioxidant and anti-inflammatory effects [27].

#### 2.8.2. Intracellular Reactive Oxygen Species Measurement

HaCaT cells were plated in 96-well plates in a 1 × 10^4^ density for 24 h. The cells were pretreated with samples for 6 h in a cell incubator and then the cell-permeable 2′,7′-dichlorodihydrofluorescein diacetate (H_2_DCF-DA; Sigma-Aldrich, St. Louis, MO, USA) was loaded and incubated for 30 min in a dark room. Consequently, the cells were washed with PBS and incubated for UVB irradiation (20 mJ/cm^2^). Immediately after irradiation, the fluorescence was recorded by SpectraMax iD3. Fluorescence with an excitation of 495 nm and an emission of 520 nm was utilized. The positive control was the same as Section 2.8.1. Cell Viability after UVB Irradiation for validating this model and procedure.

### 2.9. Statistics Analysis

The results were displayed as mean ± standard deviation. This data in this study were analyzed by one-way analysis of variance with a post hoc test of Tukey, and the analysis was conducted with SPSS v19 (SPSS Inc., Chicago, IL, USA). *p* < 0.05 was considered as a significant difference.

## 3. Results

### 3.1. Solubility Assessment of Lut

**Figure 2:**
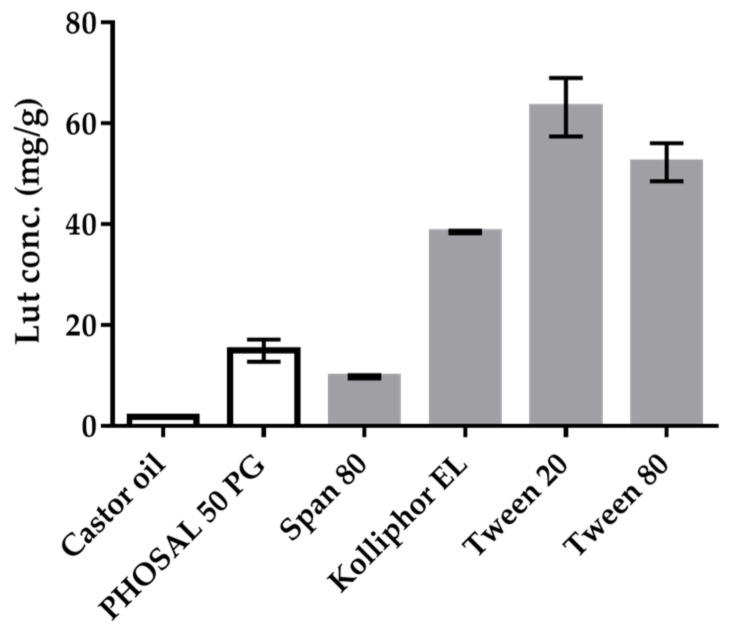
Lut solubility in castor oil and five surfactants. Data are displayed as mean ± standard deviation (*n* = 3).

Castor oil, jojoba oil, and shea butter were commonly used oils in external preparations, and the solubility of Lut in castor oil, jojoba oil, and shea butter was 1.850 ± 0.016 mg/g, 0.090 ± 0.004 mg/g, and 0.061 ± 0.004 mg/g, respectively. PHOSAL^®^ 50 PG could solubilize Lut up to 15 mg/g, which was seven times higher than that of castor oil (approximately 2 mg/g), as shown in Figure 2. Therefore, PHOSAL^®^ 50 PG was chosen as the oil phase.

The rank from highest to lowest was Tween^®^ 20 (63.21 mg/g), Tween^®^ 80 (52.33 mg/g), Kolliphor^®^ EL (38.49 mg/g), and Span^®^ 80 (9.76 mg/g). Taking safety into consideration, Kolliphor^®^ EL was chosen as the surfactant and Tween^®^ 20 as the co-surfactant.

### 3.2. Formulation Optimization

**Figure 3:**
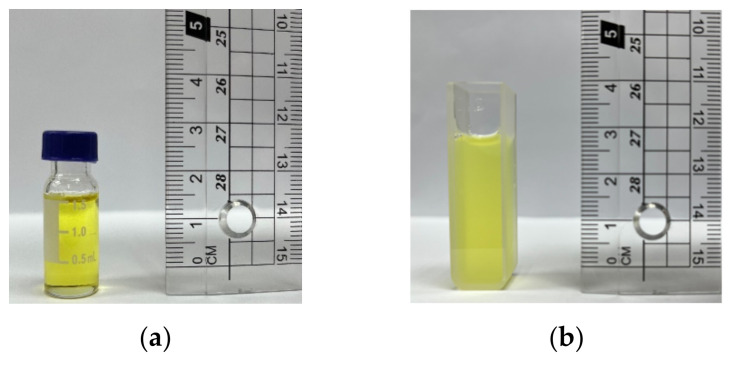
The appearance of (**a**) LSEPPs and (**b**) emulsified LSEPPs.

In this section, LSEPPs was evaluated in the emulsified status. Table 2 showed the size, polydispersity index (PDI), and zeta-potential of emulsified LSEPPs, and there were major differences among the formulations. Specifically, the droplet sizes and PDIs of F1–F4 were close to 1 μm and 0.5, respectively. On the other hand, F5–F8, which had more than 25% PHOSAL^®^ 50 PG, possessed much smaller droplets and a narrower distribution. Notably, formulations containing terpenes (F7 and F8) had significantly smaller droplet sizes with a narrow PDI. In addition, formulations with more PHOSAL^®^ 50 PG, F5–F8, needed longer emulsification time and presented a more turbid appearance after being emulsified by pH 6.4 phosphate buffer. Figure 3 displays the appearance of F7 before and after the emulsification, for example. As a result, F5–F8 were classified as grade C, which was an ideal grade for topical use in a self-emulsifying drug delivery system.

**Figure 4:**
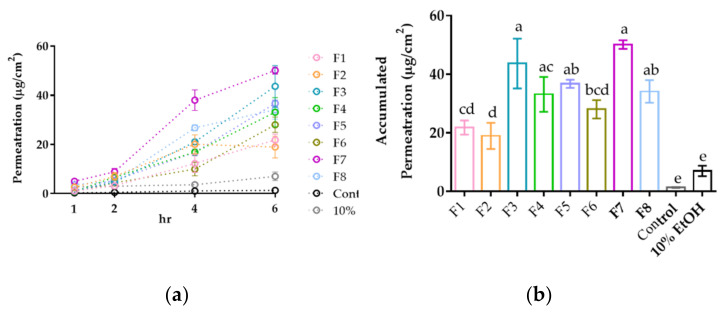
The in vitro permeability of LSEPPs presented in (**a**) each time point and (**b**) accumulated permeation at 6 h. Data are displayed as mean ± standard deviation (*n* = 3). Values that do not share the same letter were significantly different (*p* < 0.05). The composition of the control group solution is 36.8% Propylene glycol, 1.9% EtOH, and 61.3% deionized water. All groups contained 1 mg/mL Lut.

The skin PAMPA model was utilized to evaluate the capability of corneum permeability. Figure 4 illustrated the permeation profile of the designed formulations. No obvious relationship between droplet sizes and permeability was observed, which suggested that the composition played an important role in the enhancement of permeability rather than droplet sizes. The results indicated that formulations with higher ratios of PHOSAL^®^ 50 PG could bring better permeability. To prove the role of phospholipids in permeability, the composition of the control group was the same as PHOSAL^®^ 50 PG, but the phospholipid was replaced with deionized water followed by the dispersal of Lut to form a 1 mg/mL solution. The other control group was Lut (1 mg/mL) in 10% (*v*/*v*) EtOH to maintain the same concentration gradient. Obviously, the permeability of the control group was much lower than that of other formulations. The incorporation of the terpenoid as an enhancer contributed significantly to Lut permeation, and 1,8-cineole in F7 had better permeability improvement than nerolidol in F8. Therefore, F7 was chosen as the optimized formulation.

### 3.3. Physicochemical Characterization

**Figure 5:**
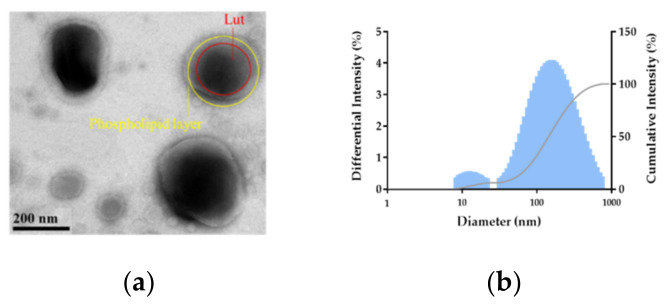
The TEM image of emulsified F7 at ×100,000 (**a**) and the droplet size distribution of emulsified F7 (**b**).

Figure 5a depicts the morphology of emulsified F7 at the scale of 200 nm. The light area illustrates the self-assembled bilayer of the phospholipid and the dense area could be related to Lut trapped in the phospholipid droplet. The characteristics of emulsified F7 were quite similar to those of non-ionic surfactant-based vesicles [28], and the droplet size and distribution of emulsified F7 measured by DLS (Figure 5b) were in accordance with those in the TEM image. Furthermore, the co-existence of spherical and oval shapes accompanied by multiple peaks in the size distribution suggested the formation of the mixed micro- and nanoemulsion system [29].

**Figure 6:**
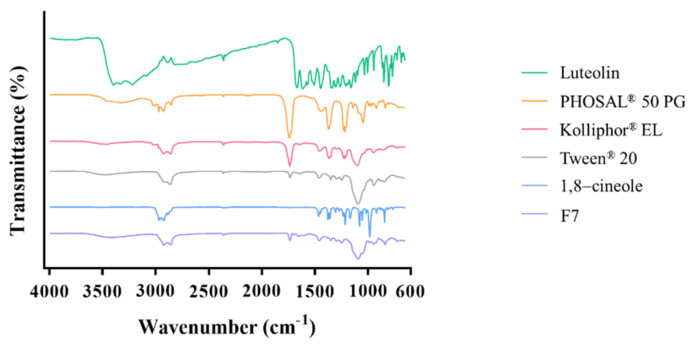
The FTIR spectrum of Lut, excipients, and F7.

FTIR was employed to investigate the compatibility between Lut and excipients from the aspect of the characterization of functional groups, as shown in Figure 6. The distinguishing peaks of Lut appeared at 3206–3533 cm^−1^ (O-H vibration), 1665 cm^−1^ (C=C vibration), 1340 cm^−1^(Phenolic-OH bending vibration), 1159 cm^−1^ (C-O-C stretching), 1033 cm^−1^, and 1002 cm^−1^(C-OH vibration) [30,31]; the characteristic peaks of PHOSAL^®^ 50 PG presented at 1736 cm^−1^ (P-O bond), 1454–1636 cm^−1^ (N-O bond), 1228 cm^−1^ (P=O bond), and 1102–1041 cm^−1^ (Amide bond conjugation) [32,33,34]; the characteristic peaks of Kolliphor EL displayed at 3474 cm^−1^ (O-H vibration), 1736 cm^−1^ (ester bond), and 1103 cm^−1^ (C-O bending) [35,36]; the characteristic peaks of Tween^®^ 20 were observed at 3477 cm^−1^ (O-H vibration), 2924–2857 cm^−1^ (C-H stretching) and 1733 cm^−1^ (O-H vibration) [37]; the distinguishing peaks of 1,8-cineole appeared at 1374 cm^−1^ (CH_3_ deformation) and 983 cm^−1^ (symmetrical bending out of the CH_2_ plane) [38]. −OH vibration and phenolic−OH bending vibration peaks of Lut and the P=O bond peak of phosphatidylcholine in PHOSAL^®^ 50 PG had shifted slightly in the F7 group, showing that possible intermolecular forces may exist. According to previous studies, it is likely that hydrogen bonding between the phosphate group of phosphosphatidylcholine and the phenolic groups of Lut occurred [34]. This interaction suggested a good match between Lut and phosphatidylcholine, allowing Lut to rigidify the phospholipid vehicle. The rigidifying effects can prevent the disorganization of the phospholipoid vehicle to achieve suitable stability when LSEPPs is emulsified [39]. The characteristic peaks of Lut were still presented. Regardless of the mild shift, there were no new peaks in the F7 group, meaning that Lut was compatible with excipients and maintaining original activity.

To mimic the realistic storage conditions of formulation, the stability of original LSEPPs was evaluated. As no water exists in the SEPPs, the excellent stability of SEPPs was expected. In this study, heating–cooling cycles, freeze–thaw cycles, and centrifugation tests were used to estimate the stability of F7. After three cycles and centrifugation, the content of Lut was measured by UV/vis spectroscopy. The amounts of Lut compared to the original were 99 ± 5%, 96 ± 4%, and 96 ± 2% in the heating–cooling cycles, freeze–thaw cycles, and centrifugation test, respectively. As expected, the loss of Lut was less than 10%, which suggested the great stability of F7.

### 3.4. Cell Viability

**Figure 7:**
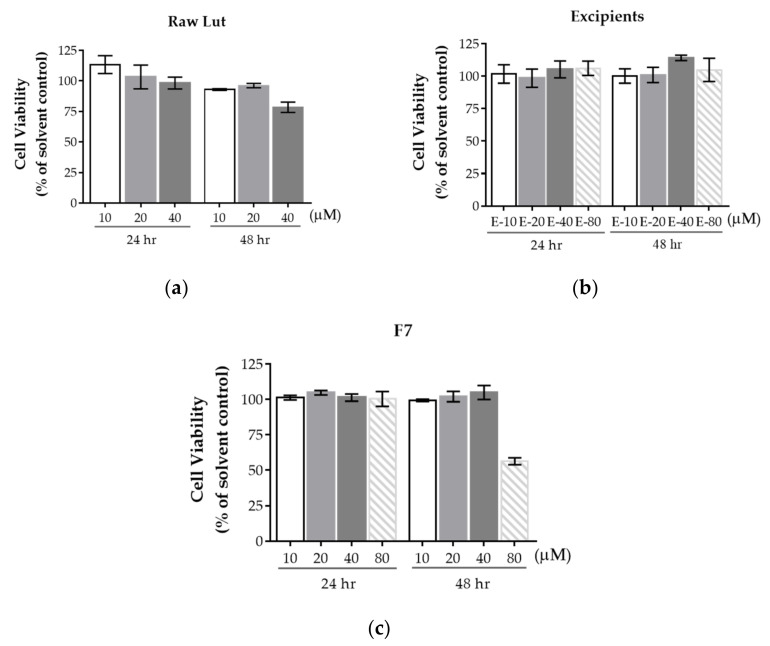
The cell viability of (**a**) Lut, (**b**) excipients, and (**c**) F7. Data are displayed as mean ± standard deviation (*n* = 3). E-10, E-20, E-40, and E-80: The equivalent amounts of excipients to reach 10, 20, 40, and 80 μM when Lut existed, respectively.

In this part, all groups were freshly dispensed to the indicated concentrations with DMEM–HG before the experiment. Figure 7 indicated the cell viability of Lut, excipients, and F7. The purpose of cell viability studies was to simultaneously estimate the safety of the formulation and to select a concentration that would not influence the results of the photoprotection assay. To ensure that the excipient would not affect cell viability, HaCaT cell viability in excipients was evaluated. In Figure 7b, the excipient did not have effects on cell viability even under 80 μM for 48 h. Lut of 40 µM mildly decreased viability after incubation for 48 h, as Figure 7a displays, while F7 improved cell viability under 40 μM for 48 h compared to the raw Lut group. In addition, the studied concentration was up to 80 μM due to the solubility improvement, as Figure 7c shows. However, F7 dramatically decreased cell viability under 80 µM for 48 h. The enhancement of the permeability and significant cellular uptake indicated the stronger driving forces of Lut into HaCaT cells, and the optimized formulation may bring more Lut into HaCaT cells [40], causing the toxicity of Lut was exhibited in such a high concentration and long incubation period.

### 3.5. Cellular Uptake

**Figure 8:**
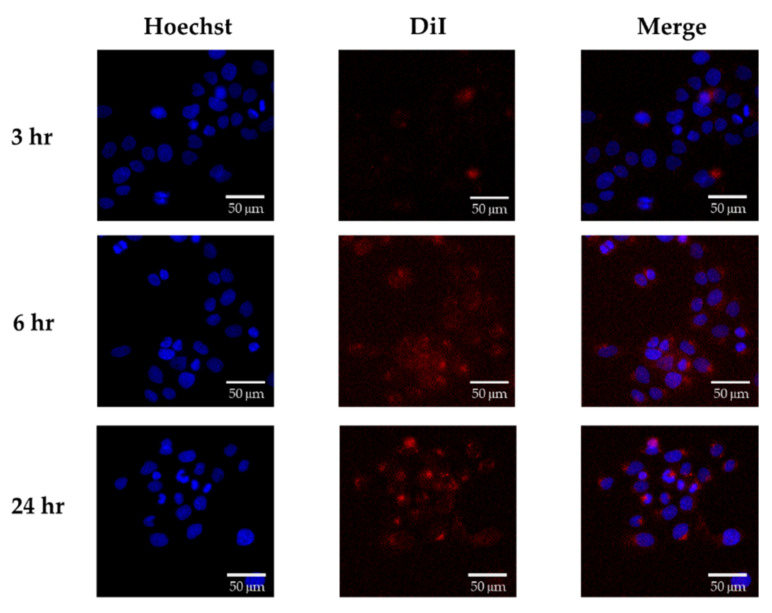
Cellular uptake of DiI-loaded F7 in HaCaT cells. Cells were incubated with DiI-loaded F7 for 3, 6, and 24 h. The fluorescent images were photographed by a widefield high-content image system (Image wells = 8).

Hoechst-stained cell nuclei presented blue fluorescence, and DiI-stained phospholipid, a composition of PHOSAL^®^ 50 PG, presented red fluorescence as shown in Figure 8. To investigate if the phospholipid droplet was an effective vehicle to promote cellular uptake, the red fluorescence of emulsified SEPPs (F7 without Lut) taken in the HaCaT cells was observed. It was obvious that DiI-loaded blank F7 had been gradually taken by cells as time went by, indicating that SEPPs could be successfully delivered into target cells.

### 3.6. Photoprotective Effects

**Figure 9:**
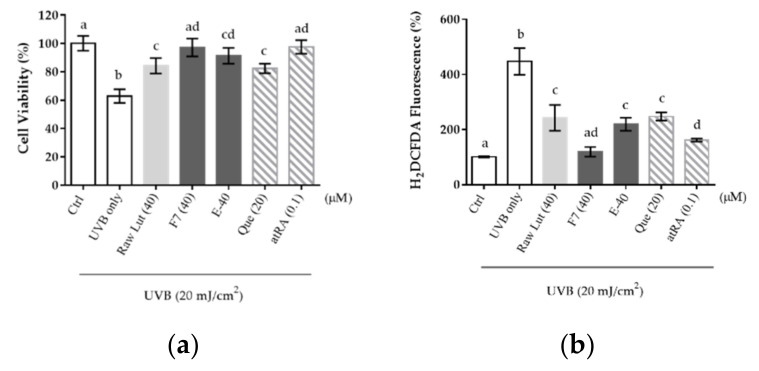
Protective effects of raw Lut, excipients, and F7 (40 μM) in HaCaT cells. Cells were pre-incubated with samples for 6 h and subjected to UVB irradiation to analyze (**a**) cell viability and (**b**) intracellular ROS. Quercetin (Que, 20 μM) and all-*trans* retinoic acid (atRA, 0.1 μM) were used as the reference control. Data were normalized with the basal group and presented as mean ± standard deviation (*n* = 3). Values that do not share the same letter were significantly different (*p* < 0.05). E-40: The equivalent amounts of excipients to reach 40 μM when Lut existed.

In this section, all groups were dispensed to indicated concentrations with DMEM 10–15 min before the study. Figure 9a presents the cell viability at 24 h after UVB irradiation (20 mJ/cm^2^), and it is obvious that each group has significantly greater cell viability compared to the viability of the UVB irradiation group (UVB only group), which means that the energy of UVB indeed resulted in injury to HaCaT cells. Lut and excipients protect cells from UVB injury, and F7 was more capable of preventing cells from UVB injury (*p* < 0.05). Compared with positive control groups, Lut and excipients of 40 μM were comparable with quercetin (Que) of 20 μM, and F7 of 40 μM was comparable with all-*trans* retinoic acid (atRA) of 0.1 μM. The same pattern was also shown in the clearance of intracellular ROS, as Figure 9b presents. The intracellular ROS value was near that of the negative control, and the effects of F7 were also more evident than the raw Lut group. Putting this together with the results in the cellular uptake study, the phenomenon suggested that the emulsified phospholipid carrier definitely can facilitate the Lut delivery into keratinocytes to scavenge the intracellular ROS.

## 4. Discussion

Phospholipids are well-established pharmaceutical excipients, which act as an emulsifier, solubilizer, and wetting agent. The structures of phospholipids vary their physicochemical properties, and this multifariousness widens the application on formulations in any administration routes [41]. In topical delivery, phospholipids increase drug–skin interactions, permeability, and the retention time on the skin surface, which makes phospholipids ideal excipients [42]. SEPPs are regarded as a lipid-based drug delivery vehicle deviated from a self-emulsifying drug delivery system, and SEPPs possess many advantages. In our study, the process of SEPP preparation does not include the risks of organic solvent residue and drying steps, and it can convert to large-scale production more easily. In addition, SEPPs can reach 100% drug entrapment with great stability [21], and the higher drug loading of SEPPs provides high thermodynamic activities, which builds up a large gradient to drive the delivery of Lut according to Fick’s first law.

Emulsified formulations with a high ratio of phospholipid (F5–F8) had smaller droplet sizes and ideal topical dispersity, which provides appropriate skin occlusion and water resistance in topical delivery [17]. Notably, formulations containing terpenes (F7 and F8) had much smaller droplet sizes than those without terpenes after emulsification. As terpenes often exist in the outer layer of phospholipid bilayers and increase the curvature of the outer layer, which leads to a smaller droplet size, the minor segment distributed in the inner layer of phospholipid would do the reverse [43].

For the in vitro permeability study, the skin PAMPA model was utilized as it is suitable to evaluate permeability among formulations in a short period of less than 24 h [44]. PAMPA is an animal-free tool to evaluate the permeability in different organs, such as skin, and this model has a high correlation (0.84) with the results obtained by human skin [45]. Zsikó et al. [46] prepared a nanostructured lipid carrier gel and compared its permeation with a Franz diffusion cell in heat separated human epidermis and a skin PAMPA model in 6 h. The permeation values of the skin PAMPA model (696.32 ± 20.50 μg/cm^2^) and human epidermis (670.85 ± 189.05 μg/cm^2^) were close, and the lower standard deviation in skin PAMPA was noted. The skin PAMPA model is regarded as a golden tool for evaluation of permeability and has been applied in the formulation screening of a nanocomplex of low molecular-weight protamine [47], ibuprofen formulations [48], and microemulgel of cannabidiol [49]. Therefore, the reliability and reproducibility of skin PAMPA are ensured. From the results, there was no definite trend that smaller droplets had better permeation, and the phenomenon could be explained by lipid–protein partition theory. Phospholipids can be integrated with cell membrane bilayers to swell the stratum corneum, which makes the corneum more fluidic and hydrated to facilitate permeation of API (e.g., Lut) through the intercellular pathway [50]. Though Lut has a high affinity with the cell membrane to accumulate on the cell surfaces due to its high degree of hydroxylation [11], the hydrogen bonding between the phenolic groups of Lut and the phosphate group of phosphatidylcholines somehow resolved the problem. Terpene is also a skin permeation enhancer as the oxygen atom of terpene could insert into the lipid bilayer of the stratum corneum to allow permeation [51], and it makes a more flexible vehicle to boost permeation [52]. Furthermore, monoterpenes (C_10_H_16_) performed much better than sesquiterpenes (C_15_H_24_), and this theory was proven in this study again as the permeation effects of 1,8-cineole are better than that of nerolidol. Some previous studies supported our findings in this study. William et al. [50] found that 1,8-cineole had the best enhancing effects for 5-FU (log P = −0.9), which was a common model drug for the establishment of permeation models. Monti et al. [53] also pointed out that 1,8-cieole was the best permeation enhancer for estradiol (log P = 4.0) in hairless mouse skin. In addition to effectiveness, the safety of 1,8-cineole was also ensured as the concentration up to 28.1% had no irritancy in humans for 24–48 h [54].

Lut is a flavonoid with low aqueous solubility, and it cannot permeate the stratum corneum easily, which limits its topical application. As SEPPs have been proven to be successfully accepted by HaCaT cells, the dramatically decreased cell viability in F7 of 80 μM at 48 h seemed reasonable, especially when raw Lut at 40 μM slightly reduced the cell viability. The composition of F7 contained 5% (*w*/*w*) 1,8-cineole, and the concentration of 1,8-cineole was equivalent to 265 µM. Such a high concentration had been proven safe in this and a previous study [55]. In the cellular uptake study, the phospholipid carrier was internalized into HaCaT cells as the negative charges on the surface and sizes smaller than 200 nm made the internalization easier [56]. Lima et al. [57] investigated internalization routes of cholesterol/L-α-phosphatidylcholine/DSPE-PEG-Mal unimellar liposomes in different cell lines, and they found that the liposome was internalized by caveolae-mediated endocytosis in human umbilical vein endothelial cells, and by micropinocytosis in human monocytes. However, the mechanisms of phospholipid internalization in HaCaT cells are still unclear as the mechanism is cell-specific and needed to be studied in the future.

In the UVB-induced cell injury models, we found that excipients also had photoprotective effects. Phosphatidylcholine could clear the overproduction of ROS, and this matched the results of the previous report [58]. According to the previous research, 1,8-cineole did not inhibit the ROS overproduction induced by UVB under 40 µM [55]. Therefore, the results may be attributed to the activities of phosphatidylcholine. Gupta et al. [59] encapsulated curcumin into liposomes, and the lipid enhanced the miscibility to escalate the antioxidative and antiaging effects of curcumin. The results in this study were in line with those of a previous study, and SEPPs improved the miscibility of Lut and amplified the photoprotective effects.

## 5. Conclusions

LSEPPs was developed and optimized in this study, and the drug loading was successfully added up to 30 mg/g. The phospholipid droplets were minimized, and the permeability was simultaneously escalated through the addition of terpenes. The optimized formulation (F7) presented desired physicochemical properties, including droplet size, distribution, dispersity grades, and permeability. The droplet was shaped in a self-assembled way after emulsification through the TEM image. In HaCaT cell models, F7 was delivered into HaCaT cells successfully and showed better photoprotective effects in the UVB-induced cell injury studies for which credit went to the synergistic effects of Lut and phospholipid.

## Figures and Tables

**Table 1:** The composition of LSEPPs.

Formulation	PHOSAL^®^ 50 PG	Kolliphor^®^ EL	Tween^®^ 20	1,8-cineole	Nerolidol
	Wt%				
F1	20	70	10	-	-
F2	20	65	15	-	-
F3	25	65	10	-	-
F4	25	60	15	-	-
F5	30	60	10	-	-
F6	30	55	15	-	-
F7	28.5	57	9.5	5	-
F8	28.5	57	9.5	-	5

-: Not applicable.

**Table 2:** The size, PDI, zeta-potential, and dispersity grade of emulsified LSEPPs.

Formulation	Size (nm)	PDI ^1^	Zeta-Potential (mV)	Dispersity Grade
F1	973.1 ± 46.1 ^a^	0.586 ± 0.017 ^a^	−10.2 ± 0.5 ^a^	B
F2	1055.9 ± 181.7 ^ab^	0.515 ± 0.020 ^ab^	−10.9 ± 0.9 ^a^	B
F3	1237.6 ± 202.6 ^a^	0.441 ± 0.040 ^b^	−11.3 ± 1.2 ^a^	B
F4	893.0 ± 81.5 ^a^	0.574 ± 0.111 ^a^	−12.2 ± 0.4 ^ab^	B
F5	253.7 ± 6.3 ^cd^	0.375 ± 0.038 ^c^	−11.9 ± 0.9 ^ab^	C
F6	459.0 ± 37.3 ^c^	0.426 ± 0.015 ^c^	−11.3 ± 0.7 ^ab^	C
F7	140.6 ± 24.2 ^d^	0.318 ± 0.015 ^c^	−12.1 ± 1.6 ^a^	C
F8	164.9 ± 13.9 ^d^	0.317 ± 0.013 ^c^	−13.4 ± 2.2 ^b^	C

^1^ PDI: polydispersity index. Data are displayed as mean ± standard deviation (*n* = 3). Values that do not share the same letter were significantly different (*p* < 0.05).

## Data Availability

Data are contained within the article.
